# Global trends in mangrove forest fragmentation

**DOI:** 10.1038/s41598-020-63880-1

**Published:** 2020-04-28

**Authors:** Dale N. Bryan-Brown, Rod M. Connolly, Daniel R. Richards, Fernanda Adame, Daniel A. Friess, Christopher J. Brown

**Affiliations:** 1 Australian Rivers Institute – Coast and Estuaries, School of Environment and Science, Griffith University, Gold Coast, QLD 4222 Australia; 2 ETH Zurich, Future Cities Laboratory, Singapore-ETH Centre, Singapore, Singapore; 3 Australian Rivers Institute, Griffith University, Nathan, QLD 4111 Australia; 4 Department of Geography, National University of Singapore, 1 Arts Link, 117570 Singapore, Singapore; 5 Australian Rivers Institute – Coast and Estuaries, School of Environment and Science, Griffith University, Nathan, QLD 4111 Australia

**Keywords:** Conservation biology, Wetlands ecology

## Abstract

Fragmentation is a major driver of ecosystem degradation, reducing the capacity of habitats to provide many important ecosystem services. Mangrove ecosystem services, such as erosion prevention, shoreline protection and mitigation of climate change (through carbon sequestration), depend on the size and arrangement of forest patches, but we know little about broad-scale patterns of mangrove forest fragmentation. Here we conduct a multi-scale analysis using global estimates of mangrove density and regional drivers of mangrove deforestation to map relationships between habitat loss and fragmentation. Mangrove fragmentation was ubiquitous; however, there are geographic disparities between mangrove loss and fragmentation; some regions, like Cambodia and the southern Caribbean, had relatively little loss, but their forests have been extensively fragmented. In Southeast Asia, a global hotspot of mangrove loss, the conversion of forests to aquaculture and rice plantations were the biggest drivers of loss (>50%) and fragmentation. Surprisingly, conversion of forests to oil palm plantations, responsible for >15% of all deforestation in Southeast Asia, was only weakly correlated with mangrove fragmentation. Thus, the management of different deforestation drivers may increase or decrease fragmentation. Our findings suggest that large scale monitoring of mangrove forests should also consider fragmentation. This work highlights that regional priorities for conservation based on forest loss rates can overlook fragmentation and associated loss of ecosystem functionality.

## Introduction

Mangroves are intertidal wetlands found along coastlines in much of the tropical, subtropical and warm-temperate world. These forests provide valuable ecosystem services including preventing erosion^1^, providing habitat for fisheries species^2^, protecting coastal communities from extreme weather events^3,4^ and storing large reserves of blue carbon, thus mitigating global climate change^5^. The services provided by mangroves are threatened by anthropogenic processes including deforestation^6^ and sea-level rise^7,8^. Historically, mangroves were subject to high rates of deforestation of up to 3.6% per annum^9^. However, since the turn of the millennium global mangrove deforestation rates have slowed, with annual loss rates of 0.2–0.7%^10,11^. Lower rates of loss are due to near total historical loss of forest patches in some regions, but also improved conservation practices^11^ and improvements in large scale monitoring techniques that provide more accurate estimates of cover and loss than were available historically^10,12^. The majority of contemporary mangrove loss occurs in Southeast Asia, where ~50% of the remaining global mangrove forest area is located, with nations such as Indonesia, Malaysia and Myanmar continuing to show losses of 0.26, 0.41 and 0.70% per year, respectively^10^.

Recently, researchers have highlighted that simply reporting mangrove total loss rates is insufficient for prioritising conservation actions^11^, if there is insufficient knowledge of the quality and spatial arrangement of habitat that remains. It is important to consider the proportional loss of mangroves, as areas with small amounts of mangrove forest will be particularly negatively affected by deforestation and resulting fragmentation, even though such small patches can still provide a disproportionate amount of ecosystem services for local populations^13^. Similarly, in addition to simply conserving mangrove forests, there is now also a focus on quantifying mangrove connectivity^14–16^. Although measurement of total areal loss is an important step towards informing conservation priorities, other metrics of environmental change, such as fragmentation, are also important indicators of habitat health^17–21^.

The ecological function and resilience of fragmented mangrove forests may be compromised in multiple ways, making fragmentation an important change to monitor^22^. For example, fragmented forests are likely to have a reduced capacity to ameliorate waves^23,24^ and so will have higher through-flow of tidal waters leading to greater erosion of sediment substrate^25^. Increased sediment erosion may affect the capacity of mangroves to accrete and keep pace with sea level rise^7,8^, so by increasing erosion fragmentation may reduce the ability of mangroves to adapt to sea level rise. In addition, increased mangrove fragmentation may mean forests are more accessible to humans, potentially leading to increased deforestation of mangroves and exploitation of species that use mangroves as habitat^26^. Finally, the biological integrity of fragmented mangroves is compromised by lower species diversity of both birds^27^ and estuarine fish^28^. Thus, the capability for mangroves to provide critical habitat for many fished species may be jeopardised by fragmentation. The biophysical impacts of fragmentation in mangroves are likely to influence the ability of forests to capture and store carbon^6,29^. Given the number of important ecological changes associated with the fragmentation of mangrove forests, we suggest that fragmentation should be explored as a way to monitor the deterioration of mangrove ecosystems at large scales.

We compared rates of mangrove fragmentation and deforestation from a high spatial resolution dataset from 2000 to 2012 at a global scale, with ~30 m resolution at the equator^10^. We used four metrics of fragmentation that represent different aspects of the quality of mangrove forests globally: clumpiness, perimeter-area fractal dimension (PAFRAC), mean patch area and the mean distance to a patch’s nearest neighbour (Supplementary Methods S1). The clumpiness index and PAFRAC assess how patches are dispersed across the landscape, and patch shape, respectively^30^. These metrics are independent of the areal extent of forests^31^, making them ideal for assessing shifts in mangrove forest arrangement. The metrics mean patch size and mean distance to nearest patch have the advantage of being immediately comprehensible and describing ecologically relevant shifts in forest arrangement^28,32^. However, these two metrics can be highly correlated with the extent of forests in the landscape^31^.

## Results

### Fragmentation

**Figure 1:**
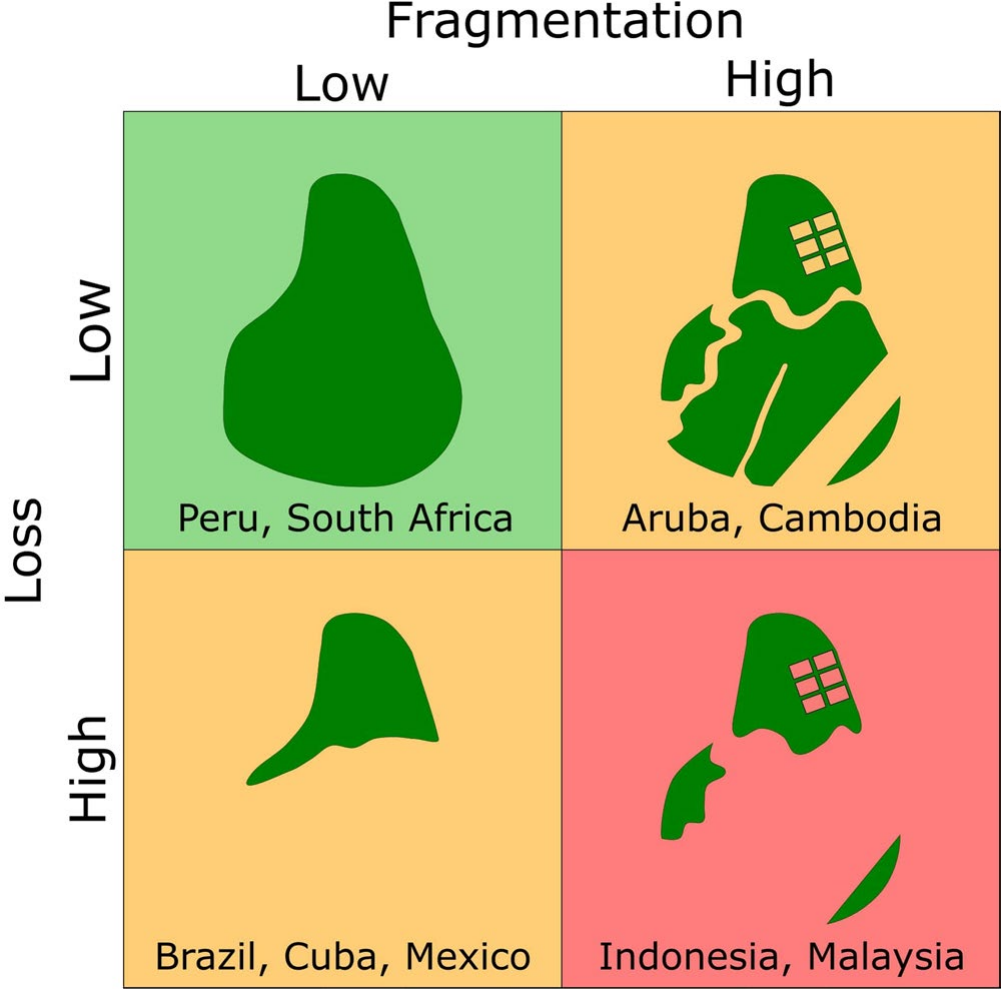
A description of similarities and disparities between fragmentation and areal loss of mangroves, with example countries.

**Figure 2:**
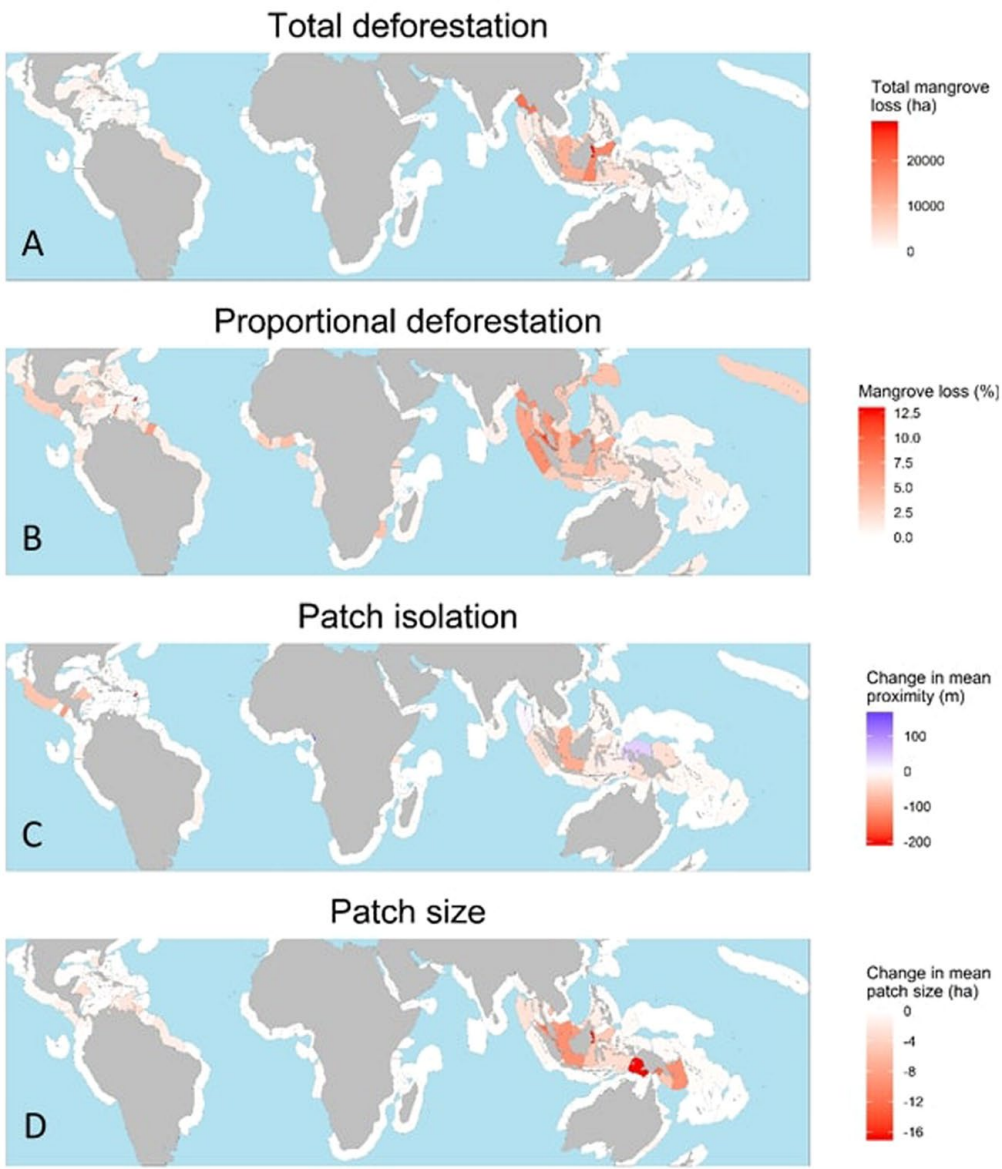
Global distribution of total mangrove loss (panel A), proportional mangrove loss (panel B) and fragmentation, measured as (1) changes in distance to nearest patch (Panel C) and, (2) shifts in mean size of mangrove patches (panel D).

**Figure 3:**
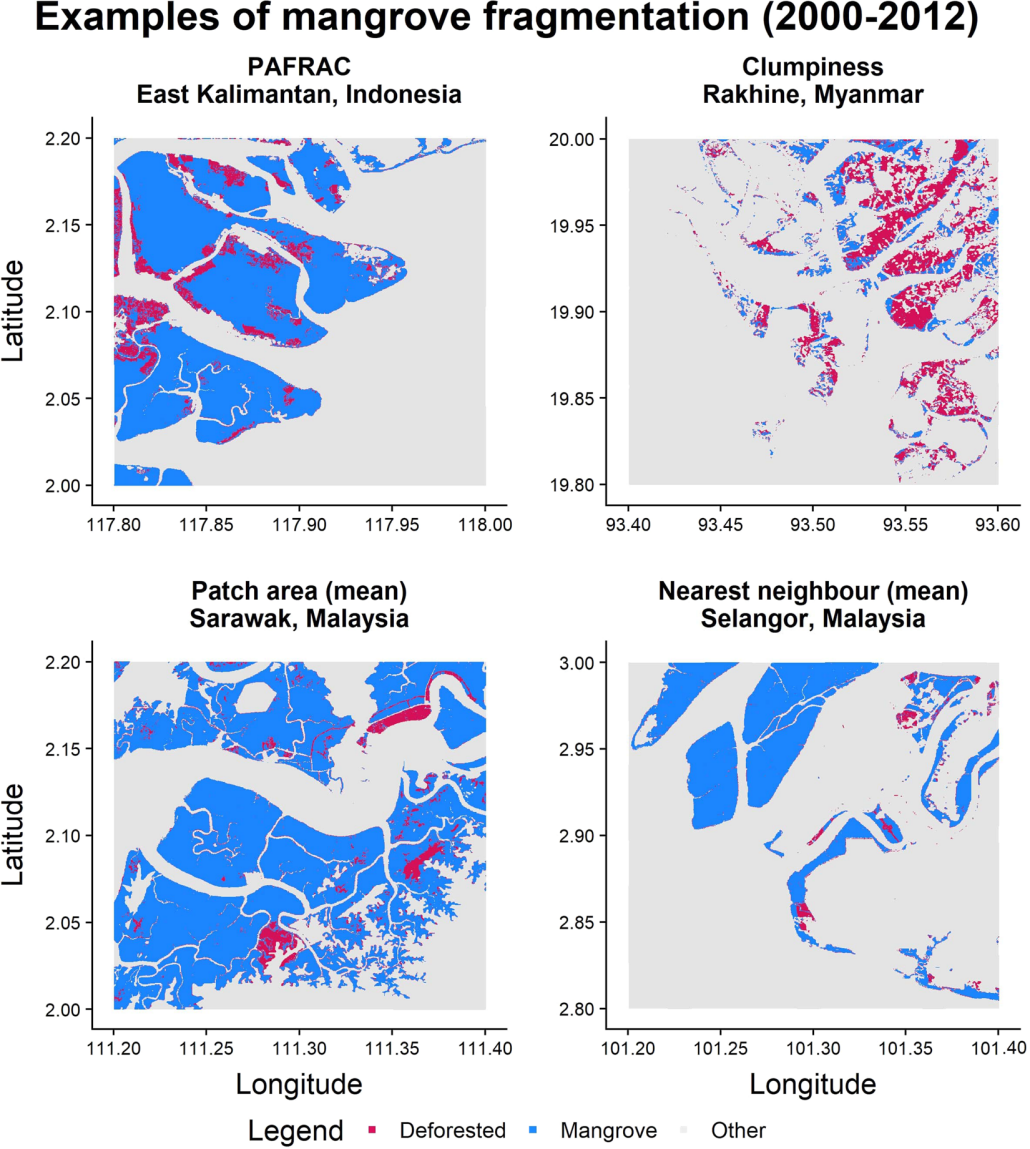
Maps of four landscapes, each demonstrating a notable shift in one of the four metrics of fragmentation employed in this study.

Broad patterns of mangrove fragmentation are related to, but distinct from, patterns in mangrove loss at the global scale. Six of the ten nations with the highest rates of mangrove loss were also in at least one of the lists for the top ten nations for fragmentation rates: Indonesia, Malaysia, Myanmar, Thailand, United States, and the Philippines (Table 1). We also identified hotspots for loss that had lower rates of fragmentation, including Brazil, northern Myanmar, Mexico and Cuba (Figs. 1, 2 and Supplementary Fig. S1). Although fragmentation is often linked to loss, there is a ubiquitous trend toward fragmentation globally, even in areas with low rates of loss (Fig. 2, Supplementary Table S1). Landscapes in regions with both high rates of loss and fragmentation, such as Myanmar, Indonesia and Malaysia, displayed high values for all measures of fragmentation (Fig. 3). Hotspots of fragmentation (within the top ten for at least two of four fragmentation metrics) include Cambodia, Cameroon, Guatemala, Honduras, Indonesia, Malaysia, New Guinea and the southern Caribbean (Aruba, Grenada, and Trinidad and Tobago). Some of these areas are associated with high deforestation rates; however, areas such as Cambodia, Cameroon, New Guinea and nations with little mangrove area in the southern Caribbean (Aruba, Grenada, and Trinidad and Tobago) have comparatively low loss rates.

**Table 1:** The top ten nations ranked by total areal loss and rates of fragmentation for each of the four main metrics. Nation and value are given.

Rank	Sum loss (km^2^)	Mean clumpiness	Mean PAFRAC	Mean patch size (Ha)	Mean nearest neighbour (m)
1	Indonesia	Aruba	Cambodia	Malaysia	St. Kitts & Nevis
	−749.90	−0.027	0.032	−7.20	−201
2	Malaysia	St. Kitts & Nevis	Aruba	Papua New Guinea	Cameroon
	−241.28	−0.022	0.027	−5.93	159
3	Myanmar	Cambodia	Malaysia	Cambodia	El Salvador
	−235.17	−0.017	0.026	−5.42	−99
4	Thailand	Japan	Trinidad & Tobago	Indonesia	Honduras
	−47.05	−0.011	0.020	−5.23	−51
5	Brazil	Thailand	Thailand	Guatemala	Kenya
	−46.34	−0.010	0.020	−5.12	−30
6	United States	Grenada	Indonesia	Cameroon	Malaysia
	−43.44	−0.010	0.018	−4.85	−24
7	Mexico	Taiwan	United States	Grenada	Indonesia
	−29.46	−0.009	0.016	−4.50	−24
8	India	Malaysia	DRC Congo	Trinidad & Tobago	Guatemala
	−27.22	−0.009	0.016	−2.85	−22
9	Cuba	Myanmar	Myanmar	Honduras	Singapore
	−26.90	−0.008	0.016	−2.51	−22
10	Philippines	Jamaica	Philippines	Venezuela	Papua New Guinea
	−26.81	−0.007	0.012	−2.29	−20

The spatial distribution of mangrove fragmentation is variable and depends on which metric of fragmentation is considered (Table 1, Fig. 2). Generally, there is a fragmentation hotspot centred in Southeast Asia, concomitant with known areas of mangrove loss^10^. There are other hotspots of fragmentation (albeit less severe than in Southeast Asia) in the Caribbean, northern South America and the eastern Pacific. These hotspots ranked highly for fragmentation in the metrics of mean distance to nearest neighbour and patch area (see Fig. 2), metrics which have high ecological relevance. Western Africa also ranked highly on the sensitive metrics of PAFRAC and clumpiness (see Supplementary Fig. S1).

### Land-use changes

**Figure 4:**
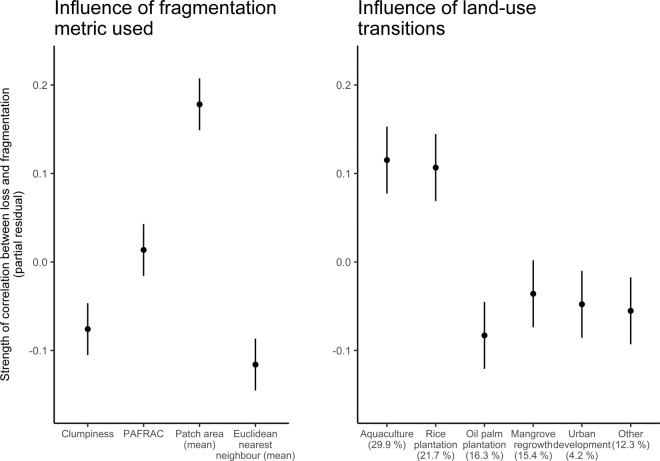
Partial effects plots for meta-analysis of Spearman rank correlations between loss and fragmentation for four fragmentation metrics and six classifications of land-use change. The partial effect sizes for (**A**) fragmentation metric and (**B**) land-use transition type are plotted relative to zero, where a value of zero indicates the average effect, positive values indicate a stronger than average correlation and negative values a weaker than average correlation. The proportion of mangrove deforestation in Southeast Asia caused by each land-use change is indicated in panel B^10^ (values do not add up to 100% due to rounding).

Fragmentation and loss were highly correlated in Southeast Asia, and this relationship was mediated by the specific land-use transition. Rank correlations indicate a strong relationship between the extent of loss and all fragmentation metrics (correlation coefficients ranged from 0.37 to 0.66, all correlations had p < 0.0001). Mean patch area was the most responsive metric to loss (rank correlation coefficient of 0.66), while the mean nearest patch was the least responsive (rank correlation coefficient of 0.37). Forests converted to aquaculture or rice plantations had the strongest correlation between fragmentation and deforestation, indicating a greater amount of fragmentation per unit area of deforestation for these types of land-use changes when compared to other types. The correlation coefficients for aquaculture and rice plantations were ~0.15 higher for these two land-uses than all others. The relationship between fragmentation and deforestation was weakest for conversion to oil palm plantations (Fig. 4).

## Discussion

We found that mangrove deforestation is often, but not always, associated with high levels of mangrove fragmentation, and the strength of this relationship varies regionally. Changes in the fragmentation metrics for the nearest patch and mean patch size can either increase or decrease when mangroves are lost. Changes in mean distance to the nearest neighbour could be caused by edges of patches being lost, single large patches being broken into multiple smaller patches or entire patches being lost. If entire patches are lost, the mean distance metric can increase or decrease, depending on the position of the remaining patches. Changes in mean patch size may be driven by either the loss of entire patches, single patches fragmenting or a reduction in patch size. Therefore, where total areal loss coincides with either positive or negative shifts in these metrics, the function of mangrove ecosystems is likely to be compromised^27,28^. Accordingly, areas identified as hotspots for either loss (such as Brazil) or fragmentation (such as Cameroon) are likely to have compromised mangrove functionality.

Southeast Asia is one of the major areas of concern for mangrove conservation because it has the highest mangrove tree species diversity^33^, the greatest areal extent of mangrove forest globally, and high deforestation rates^10^. Although mean nearest patch and mean inter-patch distance are both highly correlated to loss in this region (Supplementary Table S1) the discrepancies between the two metrics highlight how different types of land-use change might impair ecological condition differently. Distance to the nearest neighbour and patch size are important for maintaining migration corridors and patch residency, respectively, meaning that distinct drivers of deforestation (not just the extent of deforestation) may have differing effects on mangrove ecosystem functionality. For example, in landscapes dominated by mangrove regrowth (including regions with mangrove forestry), loss is highly correlated with mean patch size (*r* = 0.72) and less so for nearest mangrove forest (*r* = 0.31). Accordingly, mangrove forestry may adversely impact the capacity of a forest to harbour species, without affecting its suitability as a faunal migration corridor.

We found the correlation between deforestation and fragmentation depends on what land-use mangroves were converted to. Mangrove deforestation and fragmentation were more strongly linked for conversion to aquaculture or rice paddies than for conversion to oil-palm plantations. Conversion to oil palm plantations may occur as single continuous blocks from the landward side, often removing larger and contiguous forest patches. In contrast, conversion to rice paddies or aquaculture may occur as many smaller intrusions and thus fragment mangroves more acutely. Such piecemeal encroachment has been observed for rice paddies in the Ayeyarwady Delta, Myanmar^34^ and for aquaculture in the Mahakam Delta, Indonesia^35^.

The indirect relationship between loss, fragmentation and land-use change implies that if fragmentation and loss are considered separately management authorities may develop different conservation/restoration objectives. However, if both these degrading forces are considered in tandem, policies may shift towards encouraging/allowing different land-uses in different areas to maximise mangrove ecosystem services. Importantly, not all forms of land-use changes degrade mangroves uniformly. Mangroves converted for aquaculture can naturally recover if left undisturbed^36^, while other land-uses range between permanency (e.g. urban developments and oil palm plantations) and transiency (e.g. mangrove forestry)^37^. Future studies should seek to identify the impacts of fragmentation on mangrove ecosystems and understand how mangrove regrowth and restoration can reconnect fragmented forests.

An important caveat to the data we used was that it does not measure mangrove expansion outside of its original mapped area in 2000. Mangroves are expanding in some places^38,39^ and are being intentionally replanted in others^40^. However, over the timescale we analysed, cases of mangrove expansion and regrowth are likely to be rare relative to that of deforestation. Future studies could use new remotely sensed products, such as the Global Mangrove Watch dataset^41^, that measure both loss and growth of forests, to better understand how mangrove expansion may reconnect fragmented patches over longer timescales.

Remote sensing has facilitated large-scale mapping and the identification of conservation issues in increasingly high detail. Powerful datasets are being developed and updated regularly that allow practitioners to monitor conservation efforts with greater efficiency. Current products with large datasets of long temporal scale (such as Landsat) could be analysed using metrics or methods that assess both the areal extent and spatial arrangement of habitats. This would provide opportunities to monitor ecological degradation and the efficacy of conservation efforts in a level of detail that was previously impossible.

In this work we have identified patterns in fragmentation of mangrove forests globally, considering measures of forest extent, layout and utility to organisms. We found that areas already highlighted as hotspots of mangrove loss (Indonesia, Malaysia and Myanmar) are often associated with elevated levels of fragmentation. However, fragmentation is a far more ubiquitous form of environmental change than loss, with regional hotspots in areas otherwise considered low concern in assessments of mangrove status globally. Large-scale mangrove monitoring efforts should include fragmentation metrics and consider fragmentation alongside shifts in the area of mangrove forests. The ecological impacts of fragmentation in this intertidal habitat needs to be considered if we are to fully grasp the societal ramifications of this ubiquitous threat to mangrove health.

## Methods

### Fragmentation statistics

This study was conducted using the CGMFC-21, a freely available, Landsat derived dataset describing the global area of mangroves at 30 m resolution from 2000 until 2012^10^. The CGMFC-21 is derived from two robust and tested datasets, Hansen *et al*.’s Global Forest Change dataset^42^ and Giri *et al*.’s Global Distribution of Mangroves dataset^43^. Giri *et al*.’s dataset was an extremely important dataset for mangrove studies historically^44^ and has been used extensively^5,7,45^. However, this dataset, which offered a snapshot in time regarding the extent of mangroves in 2000, cannot be used to monitor shifts in the distribution of mangrove habitats. We chose to use the CGMFC-21^10^ for two reasons. Firstly the CGMFC-21 is the product of two robust and peer-reviewed datasets, and as such is considered a reliable source for shifts in mangrove habitats that have been used in several other studies e.g. ^5,11,46^. Secondly, the methodology for creating the CGMFC-21 was similar to the methodology utilised when identifying land-use changes in Southeast Asia^45^, thus the CGMFC-21 was extremely appropriate for working between these two datasets. The Global Mangrove Watch dataset (GMW) has recently been released, spanning the years 1996–2016 and may allow our analysis to be improved as it captures mangrove regrowth and expansion. However, we used the CGMFC-21 for this study so that results would be comparable to earlier studies of large scale mangrove loss, including the incorporated study of drivers of deforestation in Southeast Asia^10^.

Rasters for 2000 and 2012 were downloaded for analysis. A vector layer consisting of 0.2° × 0.2° cells was generated; each cell was defined as a “landscape”. Each landscape was queried to identify if mangroves were present within the borders, if mangroves were not detected the cell was removed. The geographic extent of cells that had mangroves present was then used to crop the raster images. Once the images were cropped to the appropriate extent, the image was transformed into a binary landscape. Any cell with mangrove coverage greater than zero was defined as “mangrove”. This threshold was chosen because the dataset utilised does not account for inter-annual variability within cells, with the exception of a cell losing all mangroves present (>0 to 0). Rasters were spatially transformed to the local UTM and exported as GeoTIFF files, resulting in 8,985 landscapes with mangrove presence in 2000. All spatial processing was conducted using R version 3.4.4^47^ and the packages raster^48^, rgeos^49^, rgdal^50^ and sp^51^. Fragstats^52^ was used to process the landscapes. Fragmentation statistics calculated included CLUMPY, PAFRAC, ENN_MN and AREA_MN. Total mangrove cover in the landscape was calculated using the raw cover values in the cropped raster image.

Landscapes were assigned to a nation and a biogeographical ecoregion^53^. The GADM (version 2.8) and ecoregional layers^53^ were cropped to each landscape, and the nation and ecoregion that was most dominant in the landscape were assumed to be the nation/ecoregion containing the mangroves within the landscape. The majority of landscapes were assigned only one nation (97.4%). Plotting was conducted using the R packages sf^54^ and ggplot2^55^.

### Influence of land-use transitions

For Southeast Asia, dominant land-use transitions were extracted from a previous analysis using remote sensing of Landsat imagery^45^. In the previous study, all areas of mangrove deforested in Southeast Asia between 2000 and 2012 and larger than 0.5 hectares in size were classified to identify their land cover in 2012 using a machine learning model^45^. Data on the prevalence of six types of land-use transition were extracted from this dataset: urban developments, rice paddy, oil palm plantations, aquaculture, mangrove regrowth (including mangrove forestry, rehabilitation or natural regeneration) and other (including recent deforestation with no identifiable form of land-use, deforestation caused by erosion, and conversion to non-oil palm terrestrial landscapes). Each landscape was queried for the number of mangrove patches and the total area of mangrove undergoing different land-use transitions. Many landscapes had multiple land-use transitions within their boundaries. Accordingly, the dominant land-use transition for each landscape was assigned. The land-use classification which had both; (1) the highest total area within the landscape, and (2) was present in the most (or equal to the most) mangrove patches within the landscape was considered dominant. Spearman rank correlations were conducted to identify the relationship between mangrove deforestation (loss in hectares) and absolute shifts in metrics describing habitat arrangement. The Spearman rank correlation was used because initial analyses with linear regression indicated the residuals did not conform to a normal distribution. We then modelled the correlation coefficient as a function of fragmentation metric and land-use transition using a linear model. The linear model tested the hypothesis that the extent of deforestation and fragmentation would be more strongly linked for some land-use transitions than others. All processing was conducted in R version 3.4.4^47^.

## Supplementary information

SupplementaryInformation.

## Data Availability

The datasets generated during and analysed during the current study are available in the dryad repository, WEBLINK. (To be made public upon publication).

## References

[1] Koch EW (2009). Non-linearity in ecosystem services: temporal and spatial variability in coastal protection. Front. Ecol. Environ..

[2] Nagelkerken I (2008). The habitat function of mangroves for terrestrial and marine fauna: A review. Aquat. Bot..

[3] Ouyang X, Lee SY, Connolly RM, Kainz MJ (2018). Spatially-explicit valuation of coastal wetlands for cyclone mitigation in Australia and China. Sci. Rep..

[4] Hochard JP, Hamilton S, Barbier EB (2019). Mangroves shelter coastal economic activity from cyclones. Proc. Natl. Acad. Sci..

[5] Atwood TB (2017). Global patterns in mangrove soil carbon stocks and losses. Nat. Clim. Chang..

[6] Adame MF (2018). The undervalued contribution of mangrove protection in Mexico to carbon emission targets. Conserv. Lett..

[7] Lovelock CE (2015). The vulnerability of Indo-Pacific mangrove forests to sea-level rise. Nature.

[8] Schuerch M (2018). Future response of global coastal wetlands to sea-level rise. Nature.

[9] Valiela I, Bowen JL, York JK (2001). Mangrove forests: One of the world’s threatened major tropical environments. Bioscience.

[10] Hamilton SE, Casey D (2016). Creation of a high spatio-temporal resolution global database of continuous mangrove forest cover for the 21st century (CGMFC-21). Glob. Ecol. Biogeogr..

[11] Friess DA (2019). The State of the World’s Mangrove Forests: Past, Present, and Future. Annu. Rev. Environ. Resour..

[12] Mejía-Rentería JC, Castellanos-Galindo GA, Cantera-Kintz JR, Hamilton SE (2018). A comparison of Colombian Pacific mangrove extent estimations: Implications for the conservation of a unique Neotropical tidal forest. Estuar. Coast. Shelf Sci..

[13] Curnick DJ (2019). The value of small mangrove patches. Science (80-.)..

[14] Binks RM (2019). Habitat discontinuities form strong barriers to gene flow among mangrove populations, despite the capacity for long-distance dispersal. Divers. Distrib..

[15] Hasan S, Triest L, Afrose S, De Ryck DJR (2018). Migrant pool model of dispersal explains strong connectivity of Avicennia officinalis within Sundarban mangrove areas: Effect of fragmentation and replantation. Estuar. Coast. Shelf Sci..

[16] Van der Stocken T, Carroll D, Menemenlis D, Simard M, Koedam N (2019). Global-scale dispersal and connectivity in mangroves. Proc. Natl. Acad. Sci..

[17] Herse MR, With KA, Boyle WA (2018). The importance of core habitat for a threatened species in changing landscapes. J. Appl. Ecol..

[18] Riitters KH, Wickham JD (2012). Decline of forest interior conditions in the conterminous United States. Sci. Rep..

[19] Bregman TP, Sekercioglu CH, Tobias JA (2014). Global patterns and predictors of bird species responses to forest fragmentation: Implications for ecosystem function and conservation. Biol. Conserv..

[20] Oliver TH (2015). Interacting effects of climate change and habitat fragmentation on drought-sensitive butterflies. Nat. Clim. Chang..

[21] Jacobson AP, Riggio J, M. Tait A, Baillie EM (2019). J. Global areas of low human impact (‘Low Impact Areas’) and fragmentation of the natural world. Sci. Rep..

[22] Haddad NM (2015). Habitat fragmentation and its lasting impact on Earth’s ecosystems. Sci. Adv..

[23] Dahdouh-Guebas F (2005). How effective were mangroves as a defence against the recent tsunami?. Curr. Biol..

[24] Horstman, E. M., Dohmen-Janssen, C. M., Bouma, T. J. & Hulscher, S. J. M. H. Flow routing in mangrove forests: field data obtained in Trang, Thailand. in *NCK-days 2012: Crossing borders in coastal research**: jubilee conference proceedings* 147–151, 10.3990/2.186 (University of Twente, Department of Water Engineering & Management, 2012).

[25] Thampanya U, Vermaat JE, Sinsakul S, Panapitukkul N (2006). Coastal erosion and mangrove progradation of Southern Thailand. Estuar. Coast. Shelf Sci..

[26] Barber CP, Cochrane MA, Souza CM, Laurance WF (2014). Roads, deforestation, and the mitigating effect of protected areas in the Amazon. Biol. Conserv..

[27] Li MS, Mao LJ, Shen WJ, Liu SQ, Wei AS (2013). Change and fragmentation trends of Zhanjiang mangrove forests in southern China using multi-temporal Landsat imagery (1977–2010). Estuar. Coast. Shelf Sci..

[28] Tran LX, Fischer A (2017). Spatiotemporal changes and fragmentation of mangroves and its effects on fish diversity in Ca Mau Province (Vietnam). J. Coast. Conserv..

[29] Atwood TB (2015). Predators help protect carbon stocks in blue carbon ecosystems. Nat. Clim. Chang..

[30] McGarigal, K., Cushman, S. A. & Ene, E. FRAGSTATS v4: Spatial Pattern Analysis Program for Categorical and Continuous Maps. Computer software program produced by the authors at the University of Massachusetts, Amherst. http://www.umass.edu/landeco/research/fragstats/fragstats.html (2012).

[31] Wang X, Blanchet FG, Koper N (2014). Measuring habitat fragmentation: An evaluation of landscape pattern metrics. Methods Ecol. Evol..

[32] Martin TSH (2018). Habitat proximity exerts opposing effects on key ecological functions. Landsc. Ecol..

[33] Polidoro BA (2010). The Loss of Species: Mangrove Extinction Risk and Geographic Areas of Global Concern. PLoS One.

[34] Webb EL (2014). Deforestation in the Ayeyarwady Delta and the conservation implications of an internationally-engaged Myanmar. Glob. Environ. Chang..

[35] Rahman AF, Dragoni D, Didan K, Barreto-Munoz A, Hutabarat JA (2013). Detecting large scale conversion of mangroves to aquaculture with change point and mixed-pixel analyses of high-fidelity MODIS data. Remote Sens. Environ..

[36] Proisy C (2018). Monitoring mangrove forests after aquaculture abandonment using time series of very high spatial resolution satellite images: A case study from the Perancak estuary, Bali, Indonesia. Mar. Pollut. Bull..

[37] Liao J, Zhen J, Zhang L, Metternicht G (2019). Understanding Dynamics of Mangrove Forest on Protected Areas of Hainan Island, China: 30 Years of Evidence from Remote Sensing. Sustainability.

[38] Saintilan N, Wilson NC, Rogers K, Rajkaran A, Krauss KW (2014). Mangrove expansion and salt marsh decline at mangrove poleward limits. Glob. Chang. Biol..

[39] Proisy C (2009). Mud bank colonization by opportunistic mangroves: A case study from French Guiana using lidar data. Cont. Shelf Res..

[40] Bosire JO (2008). Functionality of restored mangroves: A review. Aquat. Bot..

[41] Bunting P (2018). The Global Mangrove Watch—A New 2010 Global Baseline of Mangrove Extent. Remote Sens..

[42] Hansen MC (2013). High-Resolution Global Maps of 21st-Century Forest Cover Change. Science (80-.)..

[43] Giri C (2011). Status and distribution of mangrove forests of the world using earth observation satellite data. Glob. Ecol. Biogeogr..

[44] Heumann BW (2011). Satellite remote sensing of mangrove forests: Recent advances and future opportunities. Prog. Phys. Geogr. Earth Environ..

[45] Richards DR, Friess DA (2016). Rates and drivers of mangrove deforestation in Southeast Asia, 2000–2012. Proc. Natl. Acad. Sci..

[46] Hamilton, S. E. & Friess, D. A. Global carbon stocks and potential emissions due to mangrove deforestation from 2000 to 2012. *Nat. Clim. Chang*. 8, 240–244 (2018).

[47] R Core Team. R: A Language and Environment for Statistical Computing. (2018).

[48] Hijmans, R. J. raster: Geographic Data Analysis and Modeling. (2017).

[49] Bivand, R. & Rundel, C. rgeos: Interface to Geometry Engine - Open Source (‘GEOS’). (2017).

[50] Bivand, R., Keitt, T. & Rowlingson, B. rgdal: Bindings for the ‘Geospatial’ Data Abstraction Library. (2017).

[51] Bivand, R. S., Pebesma, E. & Gomez-Rubio, V. *Applied spatial data analysis with R*, *Second edition*. (Springer, NY, 2013).

[52] McGarigal, K., Cushman, S. & Ene, E. FRAGSTATS v4: Spatial Pattern Analysis Program for Categorical and Continuous Maps. (2012).

[53] Spalding MD (2007). Marine Ecoregions of the World: A Bioregionalization of Coastal and Shelf Areas. Bioscience.

[54] Pebesma, E. sf: Simple Features for R. (2018).

[55] Wickham, H. *ggplot2**:**Elegant* Graphics *for Data Analysis*. (Springer-Verlag New York, 2016).

