# Supplementary material for: Global trends in mangrove forest fragmentation
Source: Sci Rep. 2020 Apr 28;10:7117. doi: 10.1038/s41598-020-63880-1 (PMC7188678; doi:10.1038/s41598-020-63880-1)
Supplement: Supplementary file 1 — SupplementaryInformation. [file 41598_2020_63880_MOESM1_ESM.pdf]

Supplementary information

Global trends in mangrove forest fragmentation

Authors

Dale N. Bryan-Brown
Rod M. Connolly
Daniel R. Richards
Maria Fernanda Adame
Daniel A. Friess
Christopher J. Brown

Supplementary materials 1

All following equations and explanations are derived from the Fragstats (version 4.2) help manual.

The clumpiness index

$$Gi = \left(\frac{g_{ii}}{\sum_{k=1}^{m} g_{ik}}\right)$$

$$CLUMPY = \begin{bmatrix} \dfrac{G_i - P_i}{1 - P_i}\ for\ G_i \geq\ P_i \\ \dfrac{G_i - P_i}{1 - P_i}\ for\ G_i <\ P_i\ ;\ P_i \geq 0.5 \\ \dfrac{G_i - P_i}{-P_i}\ for\ G_i <\ P_i\ ;\ P_i < 0.5 \end{bmatrix}$$

Where g_{ii} = number of adjacencies between focal habitat and focal habitat, g_{ik} = number of adjacencies between focal habitat and non-focal habitat (m), P_i = percentage of landscape occupied by the habitat

CLUMPY ranges from -1 to1

CLUMPY = -1 is a maximally disaggregated habitat;
CLUMPY = 0 is a randomly distributed habitat;
CLUMPY = 1 is a maximally aggregated habitat;

This is a metric of like adjacencies (contagion) which is normalised by quantity of habitat in the landscape.

Perimeter-Area fractal dimension

$$PAFRAC = \frac{2}{\dfrac{\left[n_i \sum_{j=1}^{n}\left(\sum_{j=1}^{n} \ln p_{ij} \,.\, \sum_{j=1}^{n} \ln a_{ij}\right)\right] - \left[\left(\sum_{j=1}^{n} \ln p_{ij}\right)\left(\sum_{j=1}^{n} \ln a_{ij}\right)\right]}{\left(n_i \sum_{j=1}^{n} \ln p_{ij}^{2}\right) - \left(\sum_{j=1}^{n} \ln p_{ij}\right)}}$$

Where n_i is the number of patches in the landscape of habitat type i, p_{ij} and a_{ij} are the perimeter and area of patch ij, respectively.

PAFRAC ranges from 1 (patches are simple geometric shapes) and tends to 2

PAFRAC = 1; patch shape is simple
PAFRAC = 2; patch has theoretical maximal convolution

PAFRAC employs regression analysis on landscapes, accordingly only landscapes with NP>20 were considered when mapping and modelling PAFRAC in mangroves

Mean patch area

$$AREA\_MN = \frac{\sum a_{ij}}{n}$$

Where a_i is the area of patch i of habitat j (in hectares) and n is number of patches.

Mean Euclidean nearest neighbour

$$ENN\_MN = \frac{\sum \min(h_{ij})}{n}$$

Where h_{ij} is the distance in metres between patch i and j, and n is the number of patches in the landscape

Supplementary Figure S1

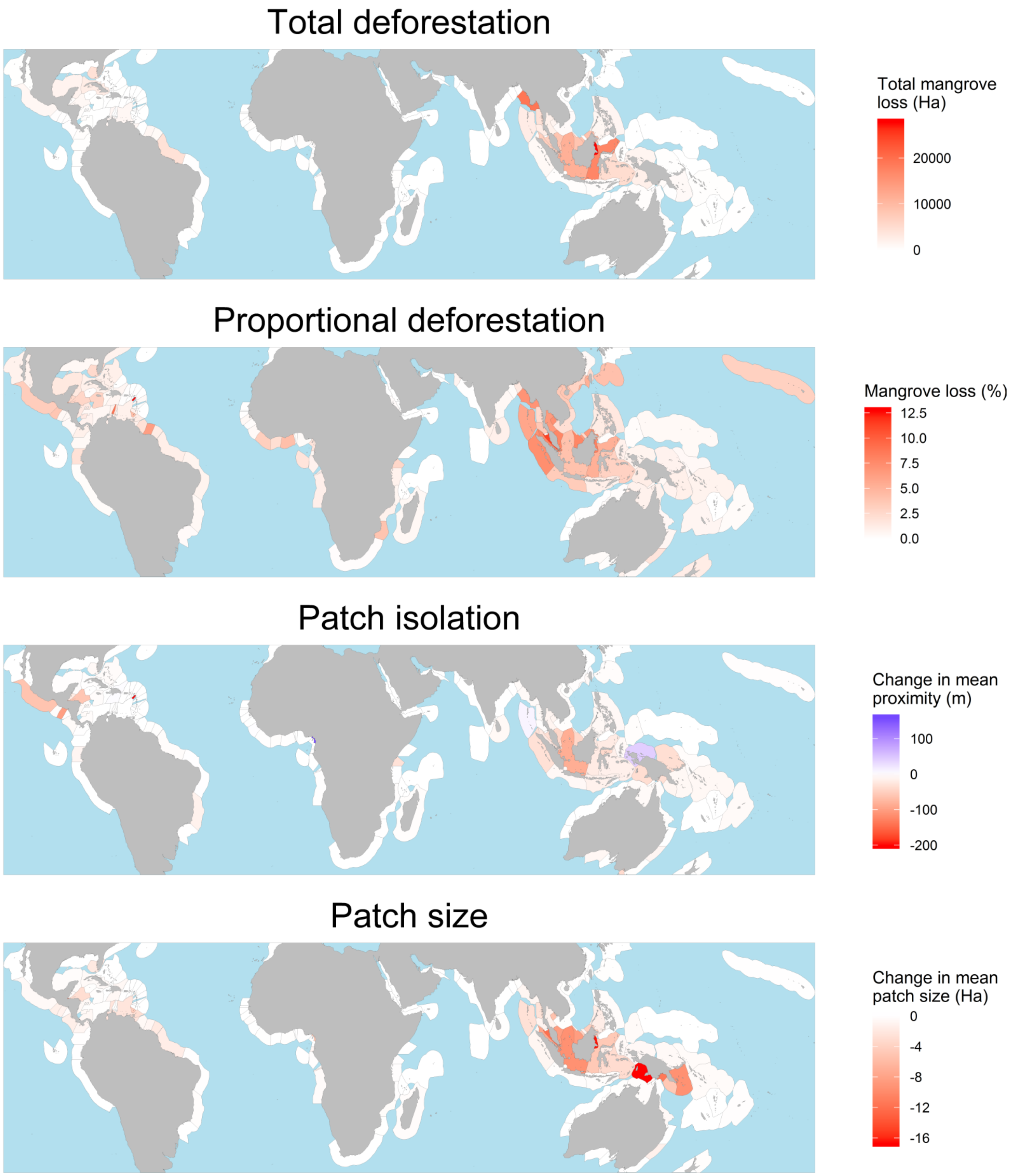

Supplementary Figure S2(a): The global distribution of shifts in the four metrics reported in the paper. Panel A) Total mangrove loss, B) Proportional mangrove loss, C) shift in the distance to nearest mangrove patch and, D) change in average size of mangrove patches.

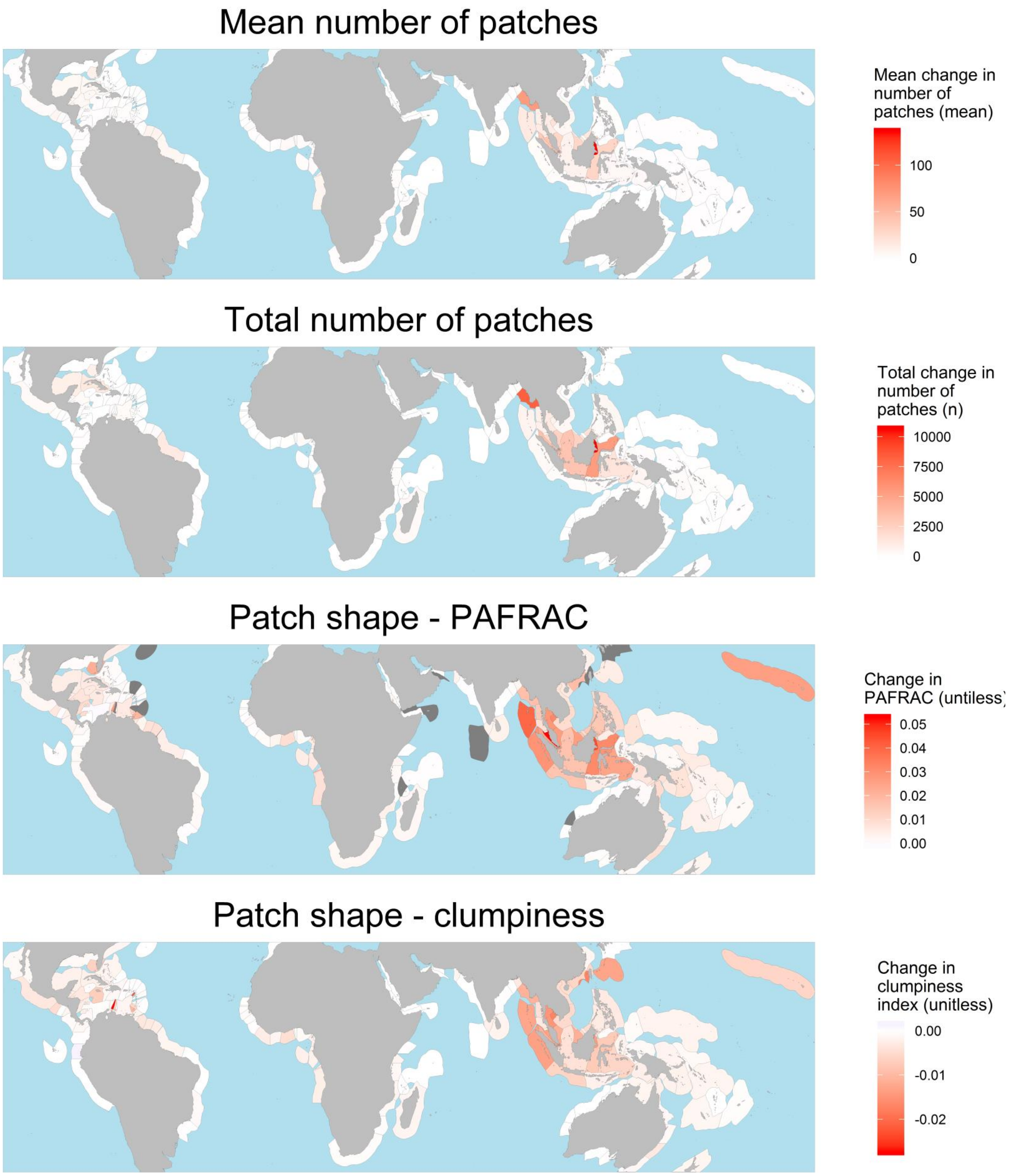

Supplementary Figure S1(b): The global distribution of shifts in the three metrics unreported in the paper. Panel A) and B) show the mean and total shift in number of patches, respectively. Panel C) and D) shows the distribution of shifts in average patch shape, as measured by PAFRAC and the clumpiness index, respectively.

Supplementary Table S1

The Spearman rank correlations (mangrove fragmentation as a function of mangrove loss) for different fragmentation metrics. Correlation coefficients are given with p-values in parentheses. Table A shows the relationship between fragmentation and loss for each land-use transition. Table B shows the relationship between fragmentation and loss without considering the dominant land-use transition of the landscapes.

Table A:

	Clumpiness index	PAFRAC	Mean patch size	Mean Euclidean nearest neighbouring patch
Aquaculture	0.53509 ($p < 0.00001$)	0.58419 ($p < 0.00001$)	0.73112 ($p < 0.00001$)	0.49822 ($p < 0.00001$)
Rice plantations	0.55937 ($p < 0.00001$)	0.68847 ($p < 0.00001$)	0.63448 ($p < 0.00001$)	0.43210 ($p = 0.00003$)
Oil palm plantations	0.25241 ($p < 0.00001$)	0.32181 ($p < 0.00001$)	0.67644 ($p < 0.00001$)	0.30532 ($p < 0.00001$)
Mangrove regrowth	0.33155 ($p = 0.00001$)	0.37602 ($p < 0.00001$)	0.72454 ($p < 0.00001$)	0.31218 ($p = 0.00005$)
Urban developments	0.34229 ($p = 0.00068$)	0.56971 ($p < 0.00001$)	0.53678 ($p < 0.00001$)	0.24801 ($p = 0.01713$)
Other	0.35591 ($p < 0.00001$)	0.37341 ($p < 0.00001$)	0.59747 ($p < 0.00001$)	0.34039 ($p < 0.00001$)

Table B

	Clumpiness index	PAFRAC	Mean patch size	Mean Euclidean nearest neighbour
All landscapes	0.38260 (1.77 E-49)	0.44830 (8.90 E-63)	0.66359 (2.08 E-176)	0.37191 (3.47 E-46)
